# ATM inhibition blocks glucose metabolism and amplifies the sensitivity of resistant lung cancer cell lines to oncogene driver inhibitors

**DOI:** 10.1186/s40170-023-00320-4

**Published:** 2023-11-06

**Authors:** Cristina Terlizzi, Viviana De Rosa, Francesca Iommelli, Antonio Pezone, Giovanna G. Altobelli, Maurizio Maddalena, Jelena Dimitrov, Caterina De Rosa, Carminia Maria Della Corte, Vittorio Enrico Avvedimento, Silvana Del Vecchio

**Affiliations:** 1 Department of Advanced Biomedical Sciences, University “Federico II”, 80131 Naples, Italy; 2 Institute of Biostructures and Bioimaging, National Research Council, Naples, Italy; 3 Department of Biology, University “Federico II”, Naples, Italy; 4 Department of Precision Medicine, University of Campania Luigi Vanvitelli, Naples, Italy; 5 Department of Molecular Medicine and Medical Biotechnology, University “Federico II”, Naples, Italy

**Keywords:** ATM, Glycolysis, OXPHOS, Oncogene-driver inhibitors, Anticancer therapy

## Abstract

**Background:**

ATM is a multifunctional serine/threonine kinase that in addition to its well-established role in DNA repair mechanisms is involved in a number of signaling pathways including regulation of oxidative stress response and metabolic diversion of glucose through the pentose phosphate pathway. Oncogene-driven tumorigenesis often implies the metabolic switch from oxidative phosphorylation to glycolysis which provides metabolic intermediates to sustain cell proliferation. The aim of our study is to elucidate the role of ATM in the regulation of glucose metabolism in oncogene-driven cancer cells and to test whether ATM may be a suitable target for anticancer therapy.

**Methods:**

Two oncogene-driven NSCLC cell lines, namely H1975 and H1993 cells, were treated with ATM inhibitor, KU55933, alone or in combination with oncogene driver inhibitors, WZ4002 or crizotinib. Key glycolytic enzymes, mitochondrial complex subunits (OXPHOS), cyclin D1, and apoptotic markers were analyzed by Western blotting. Drug-induced toxicity was assessed by MTS assay using stand-alone or combined treatment with KU55933 and driver inhibitors. Glucose consumption, pyruvate, citrate, and succinate levels were also analyzed in response to KU55933 treatment. Both cell lines were transfected with ATM-targeted siRNA or non-targeting siRNA and then exposed to treatment with driver inhibitors.

**Results:**

ATM inhibition deregulates and inhibits glucose metabolism by reducing HKII, p-PKM2^Tyr105^, p-PKM2^Ser37^, E1α subunit of pyruvate dehydrogenase complex, and all subunits of mitochondrial complexes except ATP synthase. Accordingly, glucose uptake and pyruvate concentrations were reduced in response to ATM inhibition, whereas citrate and succinate levels were increased in both cell lines indicating the supply of alternative metabolic substrates. Silencing of ATM resulted in similar changes in glycolytic cascade and OXPHOS levels. Furthermore, the driver inhibitors amplified the effects of ATM downregulation on glucose metabolism, and the combined treatment with ATM inhibitors enhanced the cytotoxic effect of driver inhibitors alone by increasing the apoptotic response.

**Conclusions:**

Inhibition of ATM reduced both glycolytic enzymes and OXPHOS levels in oncogene-driven cancer cells and enhanced apoptosis induced by driver inhibitors thus highlighting the possibility to use ATM and the driver inhibitors in combined regimens of anticancer therapy in vivo.

**Supplementary Information:**

The online version contains supplementary material available at 10.1186/s40170-023-00320-4.

## Introduction

Ataxia-telangiectasia mutated protein (ATM) is a multifunctional serine/threonine kinase involved in several signaling pathways including DNA damage response, cell cycle progression, and apoptosis by interacting with a network of substrates such as Mre11-Rad50-Nbs1 (MRN) complex, Chk2, and p53 [1–3]. ATM protein is mainly present as an inactive dimer in the nucleus where in the presence of DNA double-strand breaks, it is activated by autophosphorylation at serine 1981 and binds to substrates as a monomer. ATM can be activated even in the absence of detectable DNA damage in a MRN-independent manner in response to high levels of reactive oxygen species (ROS) [4, 5]. In this case, phosphorylated ATM is active in the form of a disulfide-linked dimer with an extranuclear localization, mainly found in the cytoplasm of neuronal cells [6] and mitochondria of myocardiocytes [7].

In addition to its role as a redox sensor, ATM was reported to be involved in glucose metabolism since it affects glucose uptake by regulating GLUT1 transport activity on cell membrane and GLUT4 translocation [8, 9]. Treatment with ATM inhibitor KU55933 abolished indeed insulin-dependent transport of glucose and caused a significant decrease of AKT phosphorylation indicating that inhibition of ATM significantly impairs insulin-mediated GLUT4 translocation. Furthermore, ATM is responsible for the metabolic diversion of glucose from glycolysis to the pentose phosphate pathway (PPP) since it modulates the enzymatic activity of glucose-6-phosphate dehydrogenase (G6PDH), the rate-limiting enzyme of the oxidative branch of PPP, showing an enhanced activity in several tumors [10]. Notably, glucose catabolism through the PPP increases the production of NADPH, a reducing agent, and ribose-5-phosphate, one of the building blocks of nucleic acid synthesis, thus providing essential elements for cellular redox homeostasis and DNA repair [11, 12].

An altered glucose metabolism is a phenotypic trait of most cancer cells that, despite the presence of oxygen, can generate energy through the glycolytic pathway rather than using oxidative phosphorylation and tricarboxylic acid cycle (TCA) [13, 14]. Oncogene-driven tumorigenesis often implies the reprogramming of glucose metabolism and the acquisition of a glycolytic phenotype that confers a growth advantage to cancer cells since a high rate of glycolysis provides a large amount of metabolic intermediates that can be used for other biosynthetic pathways such as PPP to obtain nucleotides or synthesis of amino acids and fatty acids [15–21]. In previous studies, we showed that inhibition of oncogene drivers by targeted agents caused a metabolic shift from aerobic glycolysis to oxidative phosphorylation through the concerted downregulation of hexokinase II (HKII) and pyruvate kinase M2 phosphorylated at Tyr105 (p-PKM2^Tyr105^) and upregulation of OXPHOS [22–24]. It is presently unknown whether ATM has a specific role in the acquisition or maintenance of the glycolytic phenotype in cancer cells and more importantly whether there are intersections between the oncogene-driven mitogenic pathways and ATM signaling.

The aim of the present study is to elucidate the role of ATM in the regulation of glucose metabolism in cancer cells and to test whether ATM is a suitable target for anticancer therapy. Previous studies showed indeed that FLT3-driven acute myeloid leukemia (AML) cells exposed to FLT3 inhibitors together with the inhibition of the ATM/G6PDH axis showed a higher response to therapy [25]. Furthermore, the combination of ATM inhibitor (KU55933) and EGFR tyrosine kinase inhibitor (gefitinib) showed a synergistic effect in blocking cell growth and enhancing apoptosis [26]. Based on these observations, we selected two oncogene-driven non-small cell lung cancer (NSCLC) cell lines that are resistant to EGFR inhibitors and tested whether treatment with ATM inhibitor may enhance sensitivity to EGFR inhibitors in those cells investigating the underlying molecular mechanisms.

## Materials and methods

### Cell lines and treatment

Two NSCLC cell lines were obtained from and authenticated by the American Type Culture Collection. In particular, H1993 cells are expressing high level of MET due to gene amplification (15 copy numbers) and wild-type EGFR and H1975 cells are bearing an activating point mutation in exon 21 (L858R) of the kinase domain of EGFR along with T790M secondary mutation which confers resistance to first generation EGFR inhibitors. H1993 and H1975 cells were grown in RPMI culture medium (Gibco, Thermo Fisher ATCC modification A1049101 and Gibco, Thermo Fisher, 21875091) supplemented with 10% fetal bovine serum, 100 IU/mL penicillin, and 50 µg/mL streptomycin in a humidified incubator with 5% CO_2_ at 37 °C.

Cells were treated with different targeted agents such as double mutant EGFR^L858R/T790M^ inhibitor WZ4002 (0.5 or 1 µM, Selleck Chemicals), MET inhibitor crizotinib (0.5 or 1 µM, Selleck Chemicals), and ATM inhibitor KU55933 (10 and 100 nM, Sigma-Aldrich) or vehicle for 48 h at 37 °C. Drug-induced toxicity was assessed by MTS assay (Sigma-Aldrich). Briefly, H1975 cells were plated at a density of 5000/well in 96-well plates and then treated for 48 h with increasing concentrations of KU55933 or WZ4002 (0.01–5 μM) alone and with fixed doses of KU55933 (10 nM and 100 nM) in combination with WZ4002 (0.01–5 μM). Parallel experiments were also performed in H1993 cells treated with 0.5- and 1-μM crizotinib alone or in combination with 100-nM KU55933, and results were compared to cells exposed to 100-nM KU55933 alone or to untreated controls. The optical density (OD) was measured at 450 nm using microplate spectrophotometer, after 1-h incubation with MTS at 37 °C. At least three independent assays were performed, and data are expressed as percentage of viable cells, considering the untreated control cells as 100%.

### siRNA interference

H1993 and H1975 cells were transfected with siRNA targeting ATM (sense CUUAGCAGGAGGUGUAAAU, antisense AUUUACACCUCCUGCUAAG) and control non-targeting siRNA (siCTRL) purchased from Sigma-Aldrich and used according to the manufacturer’s instructions. Briefly, H1993 and H1975 cells were plated and allowed to attach for 24 h. Then, cells were transfected with 100-nM siRNAs using the DharmaFECT reagent (Dharmacon), and after 24 h, EGFR or MET inhibitors were added for further 48 h. Finally, cells were harvested and lysed for subsequent Western blot analysis.

### Immunoblotting analysis

Untreated and treated cells were lysed on ice in RIPA lysis buffer (Sigma-Aldrich) with protease (Sigma-Aldrich) and phosphatase inhibitors (Sigma-Aldrich) and kept on ice for 30 min. The suspension was homogenized and centrifuged at 13,000 g at 4 °C for 30 min, and then supernatant containing whole cell lysates was collected.

Western blot analysis was performed by using a standard procedure. Antibodies used for Western blotting included the following: actin (A4700, Sigma-Aldrich), p-ATM^S1981^ (ab81292 Abcam), ATM (ab199726, Abcam), BIM (559685, BD Biosciences), cyclin D1 (2922, Cell Signaling Technology), α-tubulin (T9026, Sigma-Aldrich), GAPDH (5174S Cell Signaling Technology), glucose-6-phosphate dehydrogenase (sc-373886 Santa Cruz Biotechnology), hexokinase II (2867, Cell Signaling Technology), lamin A/C (2032, Cell Signaling Technology), LDH-A (3582, Cell Signaling Technology), p-p53^Ser15^ (9284S, Cell Signaling Technology), p53 (sc-126, Santa Cruz Biotechnology), p-PDH^Ser293^ (ab92696, Abcam), PDH (3205, Cell Signaling Technology), PARP (556494, BD Pharmingen), p-PKM2^Tyr105^ (3827, Cell Signaling Technology), p-PKM2^Ser37^ (11456, Signalway Antibody), PKM2 (4053, Cell Signaling Technology), vinculin (4650, Cell Signaling Technology), and a cocktail of 5 mAbs against OXPHOS (ab110411, Abcam) recognized the following proteins: 20 kD subunit of Complex I (20 kD), COX II of Complex IV (22 kD), 30 kD Ip subunit of Complex II (30 kD), core 2 of Complex III (~ 50 kD), and F1α (ATP synthase) of Complex V (~ 60 kD). A commercially available ECL kit (Advansta, San Jose, CA, USA) was used to reveal the reaction.

### Glucose consumption, pyruvate, citrate, and succinate levels in cultured tumor cells

Glucose levels were determined in conditioned media of H1993 and H1975 cells that were treated or not with KU55933. Briefly, conditioned media were removed, centrifuged at 13,000 g at 4 °C for 10 min, and then assayed for glucose concentrations using the Glucose Assay Kit (Sigma-Aldrich), following manufacturer’s instructions. Moreover, intracellular citrate, pyruvate, and succinate levels were determined in H1993 and H1975 cells using the Citrate Assay Kit (Sigma-Aldrich), Pyruvate Assay Kit (Sigma-Aldrich), and Succinate Assay Kit (Sigma-Aldrich) following manufacturers’ instructions. Briefly, cells were seeded in six-well flat-bottomed plates at a density of 3 × 10^5^ cells per well and then treated with ATM inhibitor (100-nM) for 48 h. Cells were collected and resuspended in specific substrate assay buffer and incubated with appropriate assay mix. The optical density (OD) was measured using microplate spectrophotometer, and metabolite concentrations were calculated from the corresponding standard curve and normalized to 10^6^ cells. At least three independent experiments were performed, and data were pooled.

### Glycolytic and mitochondrial ATP production

The production of ATP by glycolysis and mitochondria was determined using the Seahorse XFp Analyzer (Agilent Technologies, Santa Clara, CA, USA) and the real-time ATP rate assay kit (Agilent technologies) following manufacturer’s instructions. Briefly, H1975 and H1993 cells were seeded on cell culture miniplates and allowed to attach overnight. Cells were then treated with 10-nM and 100-nM KU55933, 1-μM WZ4002, or 1-μM crizotinib and combined therapy with 100-nM KU55933 plus 0.5-μM WZ4002 or crizotinib for 48 h. ATP production was measured after the subsequent addition of 1.5-μM oligomycin and 0.5-μM rotenone + antimycin A. Data were collected from untreated and treated cells using at least 7 independent measurements for each condition. Data were normalized for 10^6^ cells and expressed as percentage of glycolytic and mitochondrial ATP contribution.

### Statistical analysis

Statistical analysis was performed using the software MedCalc for Windows, version 12.7.0.0, (MedCalc Software, Mariakerke, Belgium). The unpaired Student’s *t*-test was used when appropriate for comparing means. ANOVA for repeated measures followed by pairwise comparisons was used to assess differences among multiple treatment groups. A value of *p* < 0.05 was considered statistically significant.

## Results

### Modulation of glycolysis and oxidative phosphorylation in response to ATM inhibitor

**Fig. 1:**
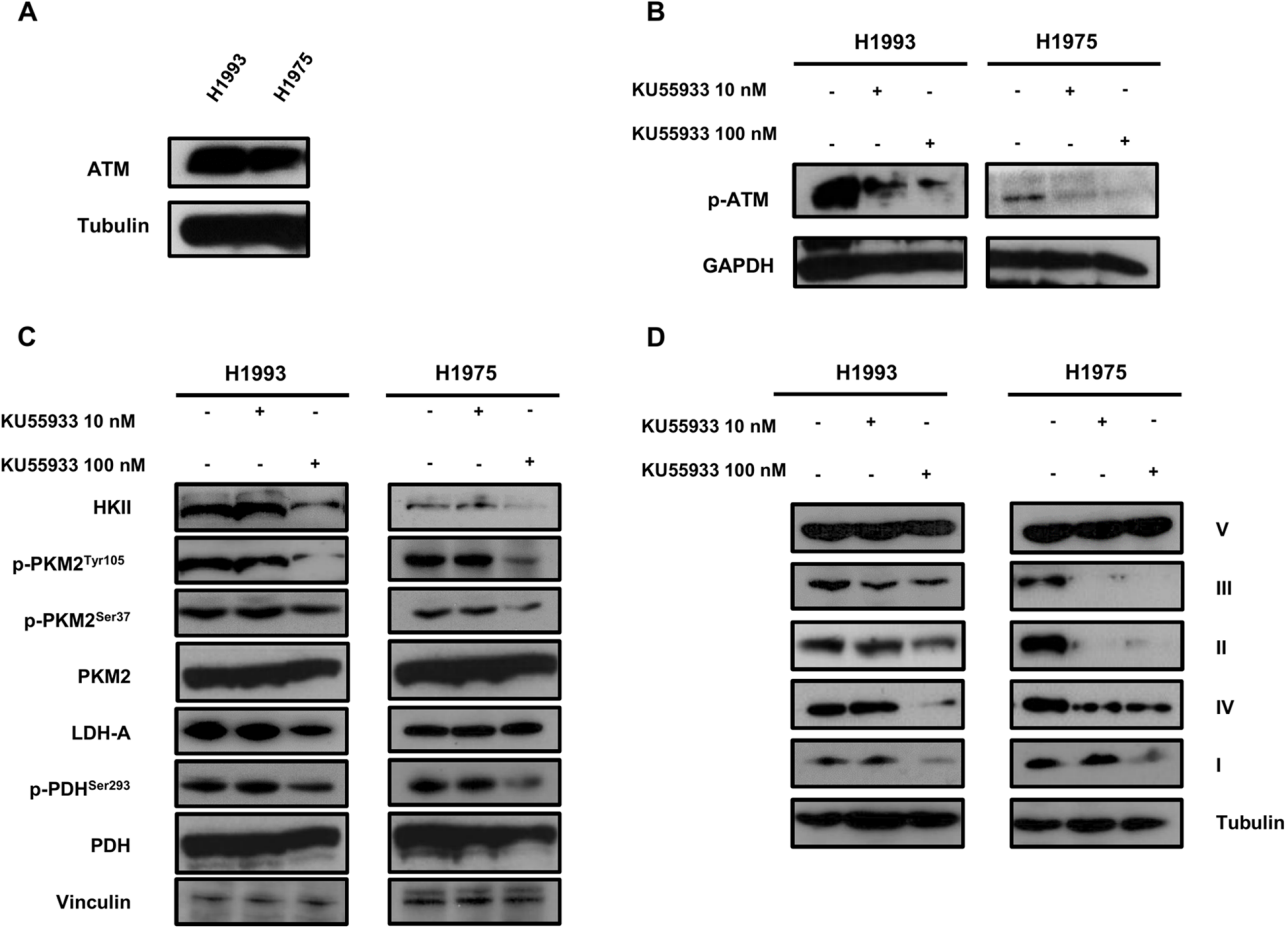
Effects of ATM inhibition on glycolysis and OXPHOS in oncogene-driven NSCLC H1993 and H1975 cells. **A** Baseline levels of ATM protein in both cell lines. **B** Inhibition of ATM phosphorylation after 48-h exposure to 10-nM and 100-nM of KU55933. **C** Levels of HKII, phospho-PKM2^Tyr105^, phospho-PKM2^Ser37^, PKM2, LDH-A, phospho-PDH^Ser293^, and PDH in basal conditions and after 48-h exposure to 10-nM and 100-nM of KU55933. **D** Levels of OXPHOS in basal conditions and after 48-h exposure to 10-nM and 100-nM of KU55933. Vinculin, GAPDH, and tubulin serve as equal loading controls

Resistant H1993 and H1975 NSCLC cells were preliminarily characterized for the expression of ATM (Fig. 1A) and then exposed to 10 and 100 nM of KU55933, an ATM-specific inhibitor for 48 h. Whole cell lysates were prepared and subjected to Western blot analysis to evaluate levels of glycolytic enzymes and mitochondrial complexes in untreated and treated cells. Figure 1B shows a dose-dependent reduction of p-ATM in both treated cell lines as compared to untreated controls. Furthermore, ATM inhibition caused a reduction of HKII, p-PKM2^Tyr105^, p-PKM2^Ser37^, and E1α subunit of pyruvate dehydrogenase complex phosphorylated at Ser293 (p-PDH^Ser293^), and these effects were more pronounced in H1975 cells compared to H1993 cells (Fig. 1C). ATM inhibition also caused a strong reduction of mitochondrial subunits. In particular, all complexes except ATP synthase (ATP5A) were reduced in H1975 cells, whereas H1993 cells showed a reduction of Complex I (NADH dehydrogenase), Complex II (succinate dehydrogenase), and Complex IV (cytochrome C oxidase) (Fig. 1D).

These findings indicate that inhibition of ATM phosphorylation strongly affects glucose metabolism by downregulating both glycolytic enzymes and OXPHOS. As expected, levels of glucose-6-phosphate dehydrogenase (G6PDH) were reduced in both cell lines exposed to ATM inhibitor (Figure S1).

### Effects of ATM inhibition on intermediates of glucose metabolism

**Fig. 2:**
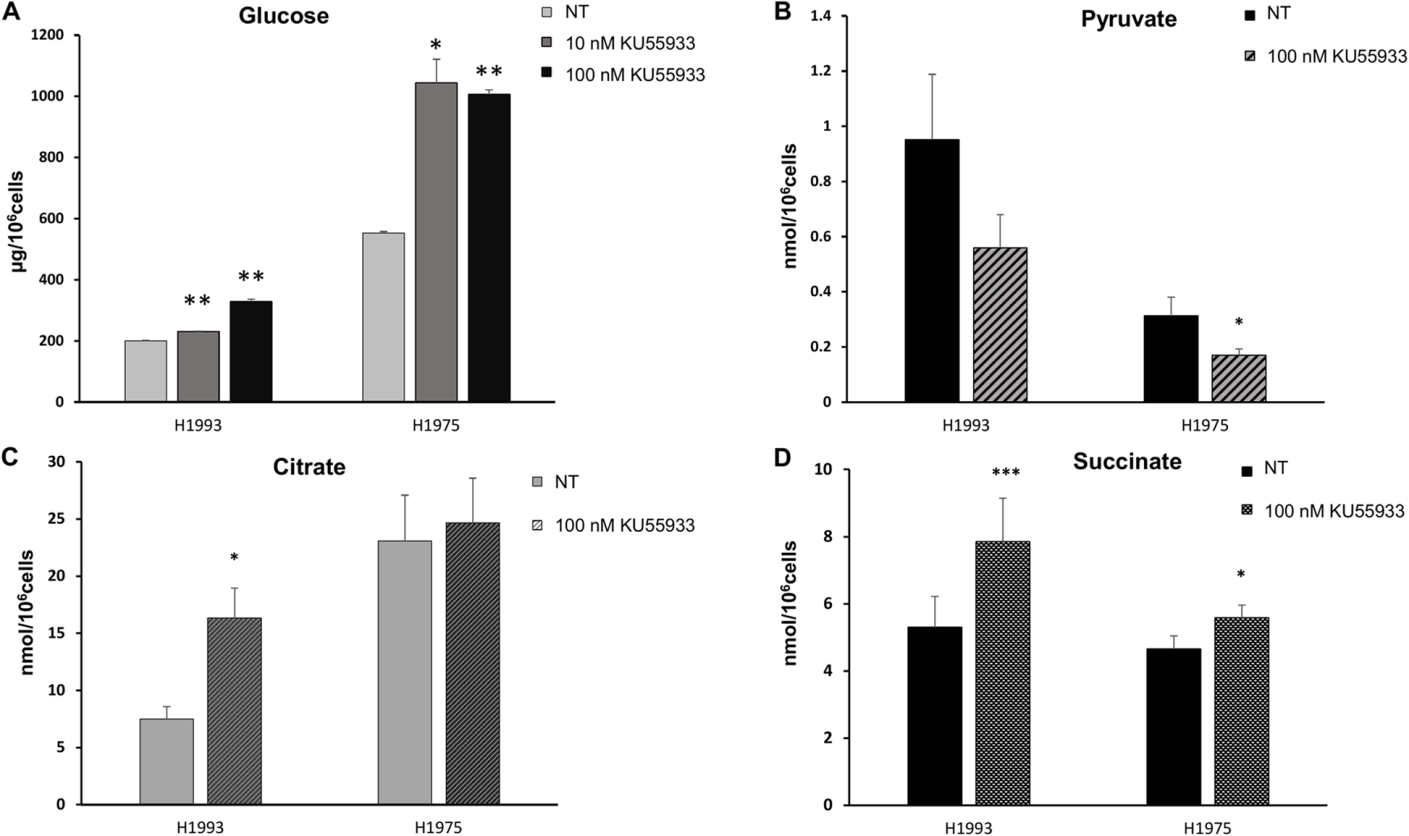
Levels of extracellular glucose and intracellular metabolic intermediates in response to ATM inhibition. **A** Levels of residual glucose in culture media of H1993 and H1975 cells exposed to 10-nM and 100-nM of KU55933 for 48 h. Levels of intracellular **B** pyruvate, **C** citrate, and **D** succinate in H1993 and H1975 cells exposed to 100-nM of KU55933 for 48 h. Symbol * indicates a *p*-value < 0.05, symbol ** indicates a *p*-value < 0.01, symbol *** indicates a *p*-value < 0.001 versus NT

To test the effects of ATM inhibition on glucose uptake and its metabolic intermediates, we tested the extracellular glucose concentration and intracellular pyruvate, citrate, and succinate levels in H1993 and H1975 cells exposed or not to KU55933 for 48 h. Extracellular glucose levels significantly increased after treatment with ATM inhibitor in both cell lines indicating a reduction of glucose consumption in treated cells compared to untreated controls (Fig. 2A). This effect was more prominent in H1975 cells as compared to H1993 cells. Levels of intracellular pyruvate decreased in both cell lines after therapy with KU55933 reaching statistical significance only in H1975 cells (Fig. 2B). Citrate levels were significantly increased after therapy in H1993 cells, whereas they were only slightly increased in H1975 cells (Fig. 2C). Furthermore, succinate concentrations were significantly increased in both cell lines, more markedly in H1993 cells (Fig. 2 D).

These findings along with results of Fig. 1 indicate that inhibition of ATM by downregulating glycolytic enzymes reduces the consumption of glucose from the medium and consequently decreases pyruvate levels. On the other hand, inhibition of ATM downregulates also mitochondrial complexes, and hence, energy metabolism is greatly impaired in both cell lines. In the absence of an abundant energy source, it is likely that replenishment of tricarboxylic acid cycle (TCA) intermediates such as citrate and succinate can be provided by additional metabolic substrates from other biosynthetic pathways such as glutamine, amino acids, or fatty acids.

### Silencing of ATM protein by small interfering RNA

**Fig. 3:**
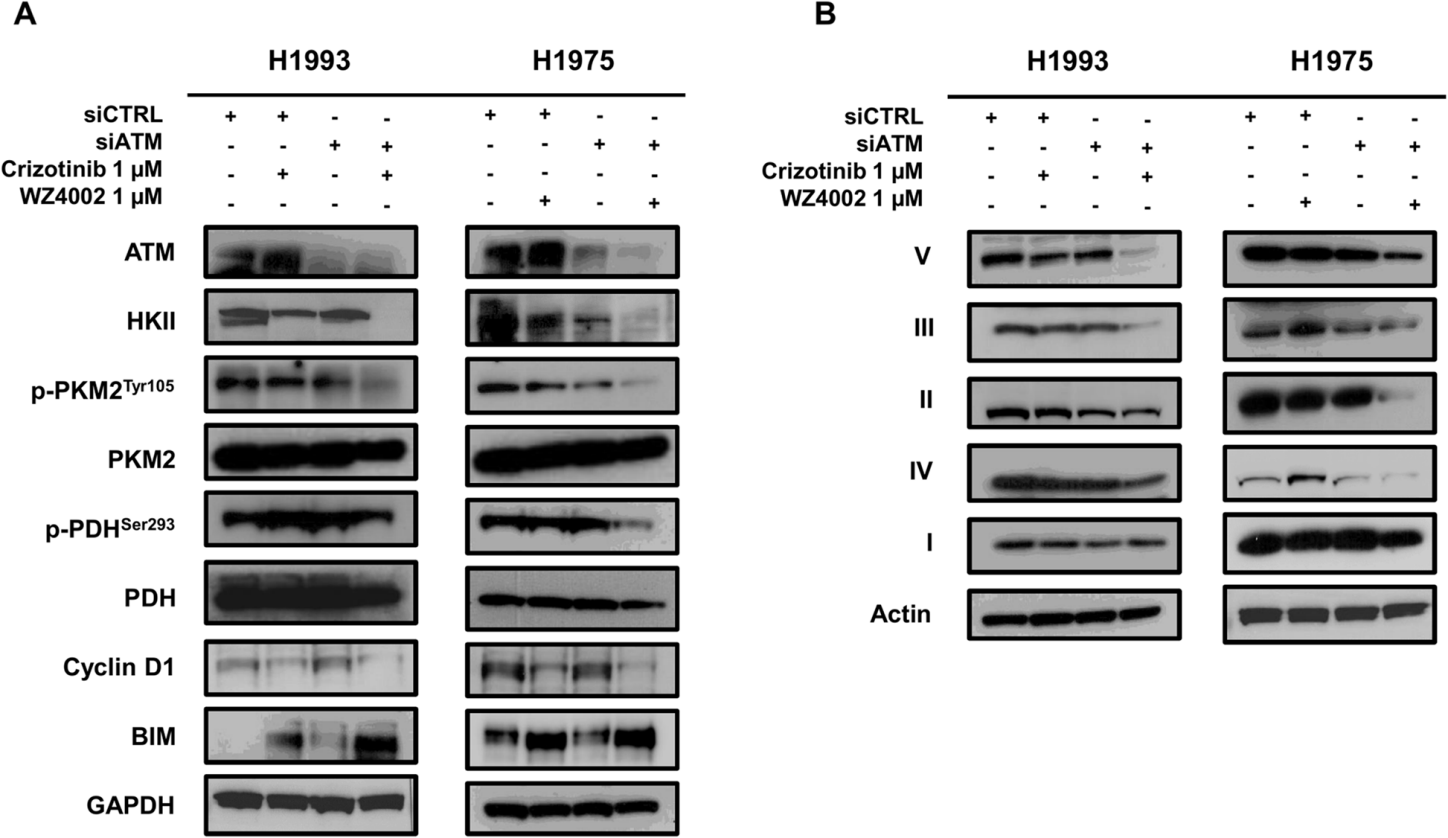
Effects of ATM silencing in oncogene-driven H1993 and H1975 cells. **A** Levels of HKII, phospho-PKM2^Tyr105^, PKM2, phospho-PDH^Ser293^, PDH, cyclin D1, and BIM in H1993 and H1975 cells transfected with ATM-targeted small interfering RNA or siRNA control and exposed to crizotinib or WZ4002 for 48 h. **B** Levels of OXPHOS in H1993 and H1975 cells transfected with ATM-targeted small interfering RNA or siRNA control and exposed to crizotinib or WZ4002 for 48 h. GAPDH and actin serve as equal loading controls

To test the effects of downregulation of ATM, both cell lines were transfected with non-targeting (siCTRL) and ATM-targeted siRNA (siATM). As shown in Fig. 3A, a strong decrease of ATM was observed in both cell lines transfected with ATM-targeted siRNA. Downregulation of ATM caused a reduction of HKII and p-PKM2^Tyr105^ in both cell lines as well as a slight decrease of mitochondrial Complex IV and III in H1975 cells (Fig. 3A and B). When exposed to selective driver inhibition, ATM-silenced cells showed a decrease of HKII, p-PKM2^Tyr105^, and p-PDH^Ser293^ that was higher than in siCTRL cells exposed to the same agent. Furthermore, a stronger reduction of cyclin D1 and a higher upregulation of BIM were observed in both ATM-silenced cell lines as compared to siCTRL cells exposed to crizotinib or WZ4002. Interestingly, the same potentiation effect between ATM silencing and driver inhibition was observed when testing levels of OXPHOS in treated cells. In fact, the simultaneous downregulation of ATM and driver inhibition caused a reduction of levels of Complexes II, III, IV, and V as compared to those found in cells exposed to each treatment alone (Fig. 3B).

#### Effects of combined treatment with ATM and driver inhibitors in H1975 and H1993 cells

**Fig. 4:**
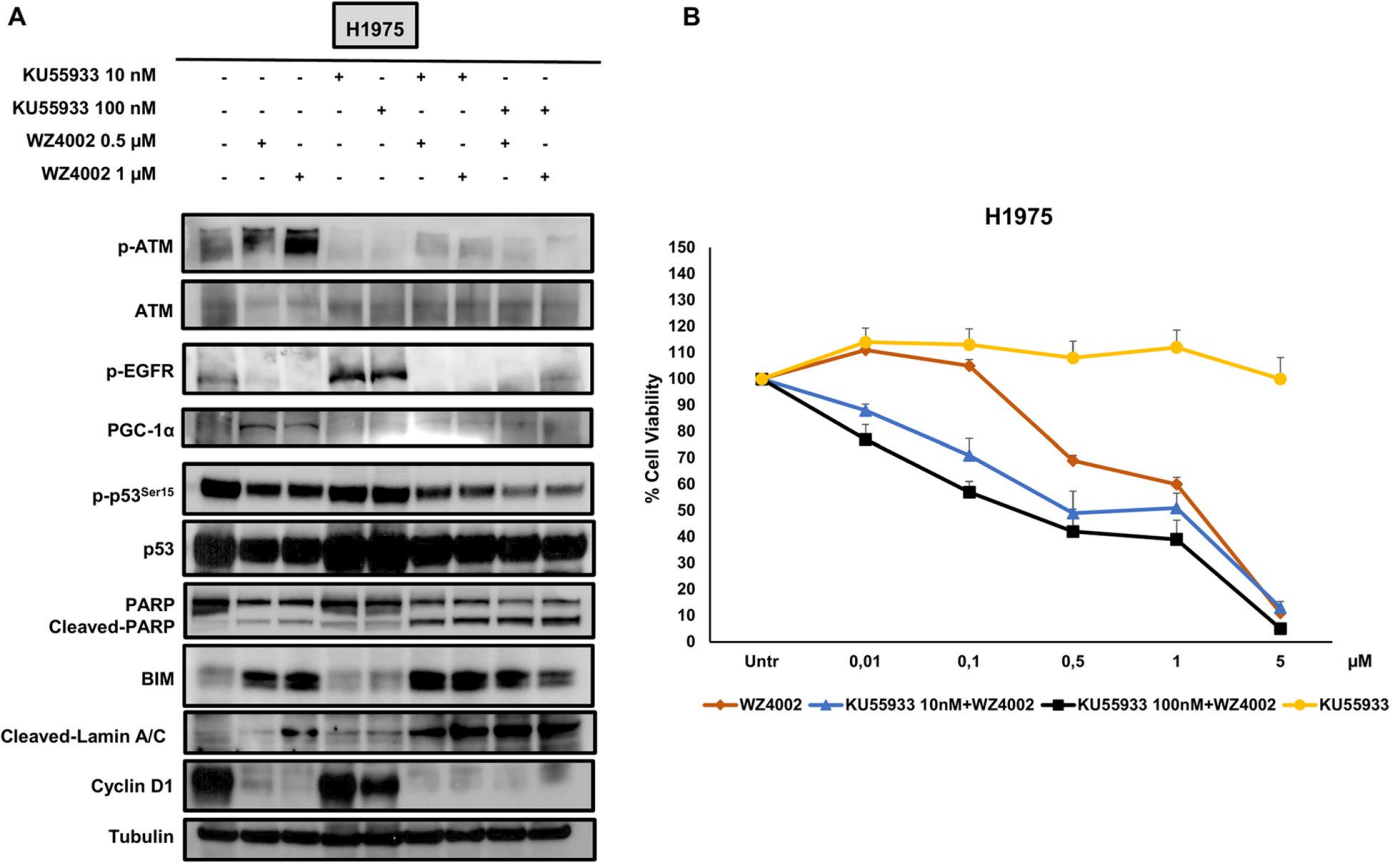
Combined treatment with KU55933 and WZ4002 in H1975 cells. **A** Levels of p-ATM, ATM, p-EGFR, PGC-1α, p-p53, p53, PARP and cleaved PARP, BIM, cleaved-lamin A/C, and cyclin D1 in H1975 treated with KU55933 (10-nM and 100-nM), WZ4002 (0.5 μM and 1 μM), and their combination for 48 h. **B** Cell viability assay performed in H1975 cells exposed to increasing concentration of KU55933 (0.01–5 μM) or WZ4002 (0.01–5 μM) alone and to combination treatment with a fixed concentration of KU55933 (10-nM and 100-nM) plus increasing doses of WZ4002 (0.01–5 μM). Tubulin serves as equal loading control

H1975 cells were treated with KU55933 (10-nM and 100-nM) and WZ4002 (0.5 μM and 1 μM) alone or in combination for 48 h. Expression levels of p-ATM, ATM, p-EGFR, PGC-1 α, p-p53^Ser15^, p53, PARP and cleaved PARP, BIM, lamin A/C, and cyclin D1 were evaluated by Western blot analysis (Fig. 4A). Notably, p-ATM levels along with PGC-1α expression were increased by the exposure to WZ4002, whereas p-EGFR, p-p53, and cyclin D1 showed a reduction after the same treatment. As expected, treatment with WZ4002 alone caused a strong upregulation of BIM and a slight increase of cleaved PARP and cleaved-lamin A/C. Treatment with KU55933 alone caused a dramatic reduction of p-ATM along with a slight decrease of cyclin D1 only at the highest dose. Furthermore, levels of p-p53^Ser15^, p53, and BIM remained unchanged, whereas level of p-EGFR increased, and cleaved PARP showed a minimal increase. Combined treatment with KU55933 and WZ4002 showed a cumulative dose-dependent decrease of p-p53^Ser15^ that was more pronounced as compared to those obtained with single agent alone. Notably, combination of low doses of KU55933 (10-nM) with WZ4002 showed a strong upregulation of BIM, increased levels of cleaved PARP and cleaved-lamin A/C, and a reduction of cyclin D1 that were higher than those obtained with single agent alone. Similar results were obtained with combination of high doses of KU55933 (100-nM) with WZ4002, although at the highest doses of KU55933 and WZ4002 the potentiation effect was less evident for levels of BIM and cyclin D1. Then, H1975 cells were treated for 48 h with increasing concentrations of KU55933 or WZ4002 (0.01–5 μM) alone and with fixed doses of KU55933 (10-nM and 100-nM) in combination with WZ4002 (0.01–5 μM) and were subject to cell viability assay (Fig. 4B).

In agreement with previous findings, treatment with increasing concentrations of KU55933 alone did not cause any significant change in cell viability, whereas WZ4002 alone decreased cell viability showing an IC50 > 1 μM. Combined treatment with KU55933 (10-nM) and WZ4002 caused a stronger reduction of cell viability as compared to that obtained with WZ4002 alone showing an IC50 of approximately 0.5 μM. In particular, 0.01 and 0.1 μM of WZ4002 in combination with 10-nM KU55933 showed a statistically significant reduction of cell viability (*p* < 0.005) as compared to treatment with WZ4002 alone. The addition of higher doses of KU55933 (100-nM) to the same concentration of WZ4002 did not show a further potentiation of WZ4002 effects. These findings indicate that KU55933 at very low doses is able to enhance the effects of EGFR TKIs in oncogene driven H1975 cells and can be used in combined treatment regimens without causing toxic effects.

**Fig. 5:**
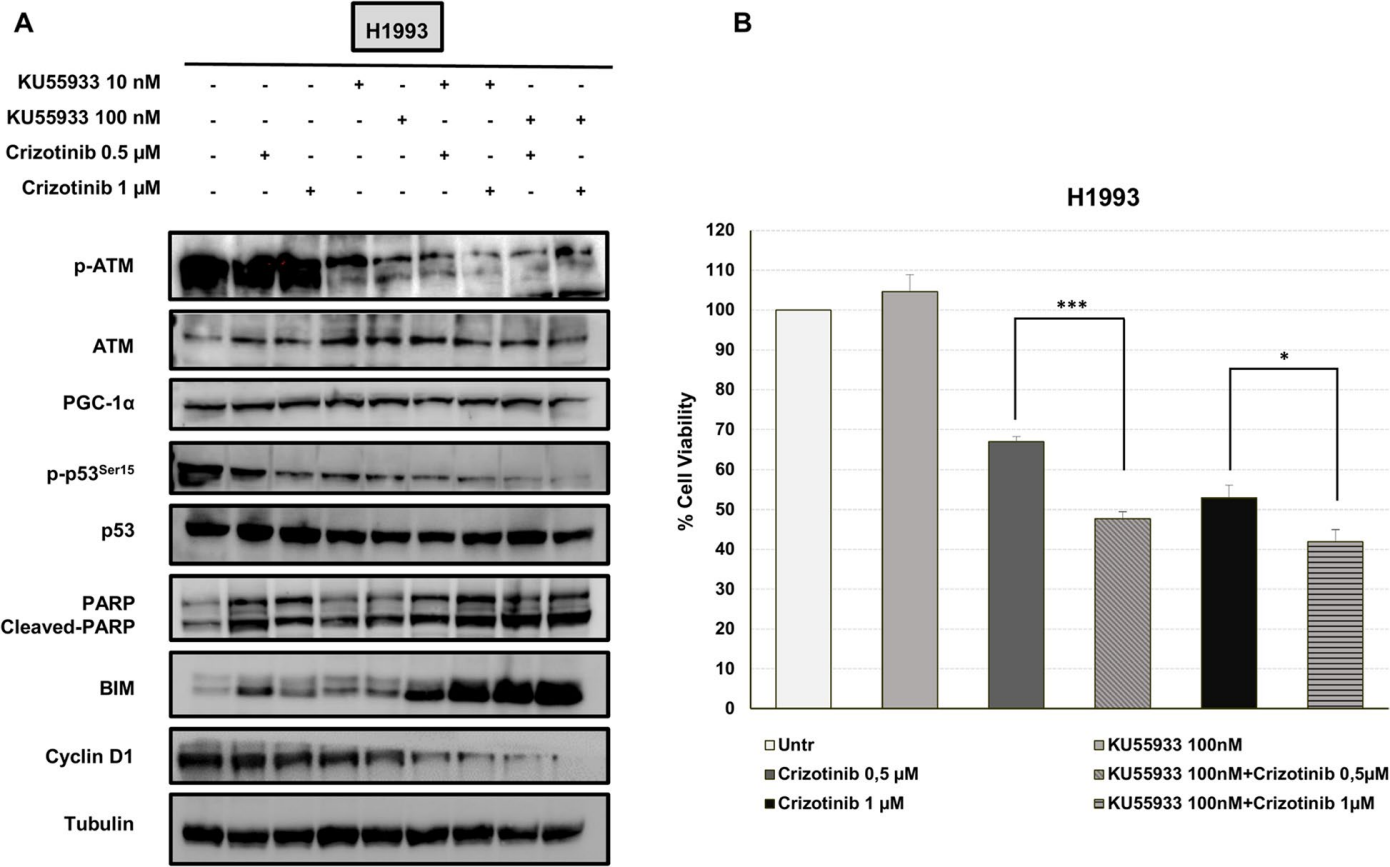
Combined treatment with KU55933 and crizotinib in H993 cells. **A** Levels of p-ATM, ATM, PGC-1α, p-p53.^Ser15^, p53, PARP and cleaved PARP, BIM, and cyclin D1 in H1993 treated with KU55933 (10- nM and 100-nM), crizotinib (0.5 μM and 1 μM), and their combination for 48 h. **B** Cell viability assay performed in H1993 cells exposed to KU55933 (100-nM), crizotinib (0.5 μM–1 μM), and their combination. Tubulin serves as equal loading control. Symbol * indicates a *p*-value < 0.05. Symbol *** indicates a *p*-value < 0.001

In parallel experiments, H1993 cells were treated with KU55933 (10-nM and 100-nM) and crizotinib (0.5 μM and 1 μM) alone or in combination for 48 h. In Fig. 5A, the combined treatment caused a strong reduction of cyclin D1 along with a dramatic upregulation of BIM and cleaved PARP indicating a potentiation effect on both proliferation and apoptosis as compared to single-agent treatment. Accordingly, the addition of KU55933 (100-nM) to crizotinib at 0.5 or 1 μM caused a significant decrease of cell viability as compared to treatment with crizotinib alone (Fig. 5B).

### Glycolytic and mitochondrial ATP production

**Fig. 6:**
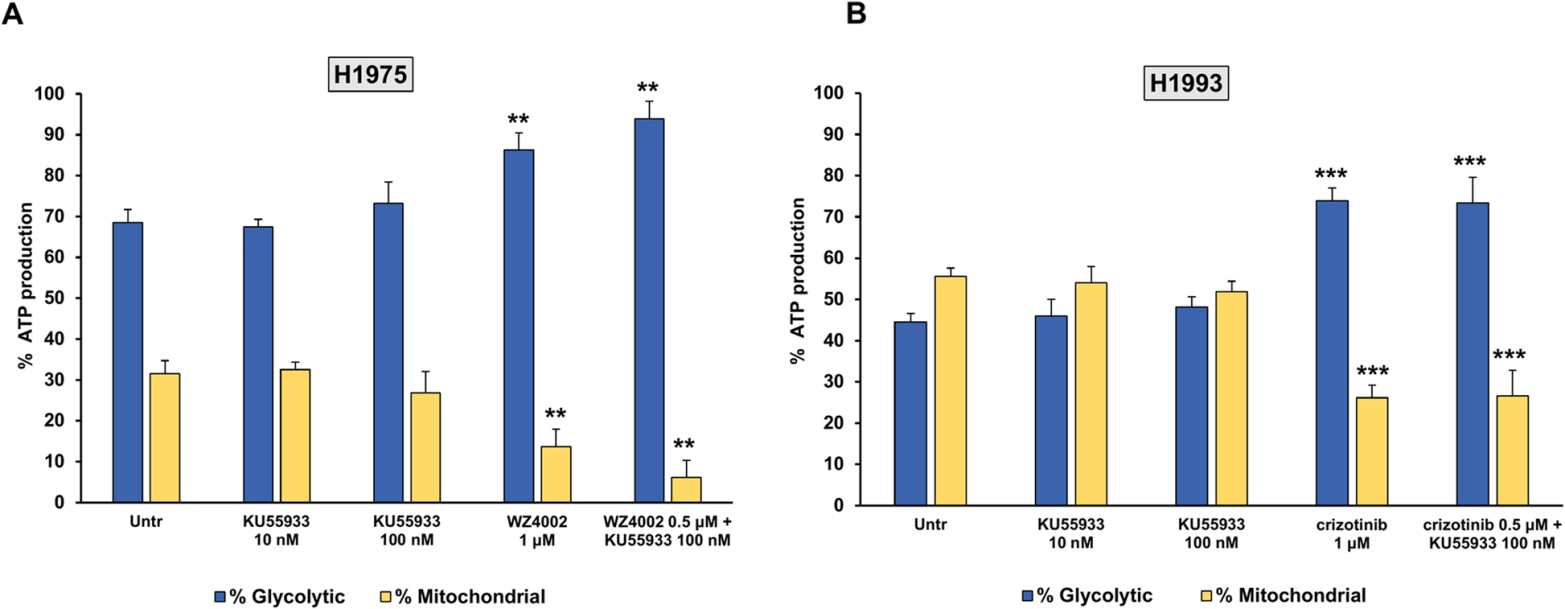
Percentages of glycolytic and mitochondrial ATP production in untreated and treated H1975 and H1993 cells. **A** H1975 cells were treated for 48 h with 10-nM and 100-nM KU55933, 1-μM WZ4002, and a combination of 100-nM KU55933 with 0.5-μM WZ4002. **B** H1993 cells were treated for 48 h with 10-nM and 100-nM KU55933, 1-μM crizotinib, and a combination of 100-nM KU55933 with 0.5-μM crizotinib. Compared to untreated cells, the symbol ** indicates *p* < 0.01, whereas *** indicates *p* < 0.001

In order to test the effects of different treatments on ATP production, the percentages of glycolytic and mitochondrial ATP levels were determined in H1975 and H1993 cells exposed to treatment with KU55933 alone or in combination with oncogene inhibitor, and results are shown in Fig. 6. A statistically significant reduction of mitochondrial ATP production (*p* < 0.01) was observed in H1975 cells treated with 1-μM WZ4002 or a combination of 0.5-μM WZ4002 and 100-nM KU55933 as compared to untreated cells (Fig. 6A). The same treatments caused a concomitant significant increase of glycolytic ATP production (*p* < 0.01) in H1975 cells. Notably, the addition of KU55933 to half-dose of W4002 in the combination regimen caused a further reduction of mitochondrial ATP production as compared to W4002 alone, although such difference did not achieve statistical significance. Similarly, a statistically significant reduction of mitochondrial ATP production (*p* < 0.001) and a significant increase of glycolytic ATP levels (*p* < 0.001) were observed in H1993 cells treated with 1-μM crizotinib or a combination of 0.5-μM crizotinib and 100-nM KU55933 as compared to untreated control (Fig. 6B).

## Discussion

Our study showed that exposure of NSCLC cells to low doses of ATM inhibitor induces a combined downregulation of glycolytic enzymes and OXPHOS with a significant reduction of glucose consumption and energy supply. Despite the decrease of glucose-dependent energy production, NSCLC cells exposed to ATM inhibitor do not activate the apoptotic program and survived as shown by MTS assay suggesting the exploitation of alternative energetic substrates. Nevertheless, the combined treatment of NSCLC cells with ATM and driver inhibitors strongly enhanced the cytotoxic effects of oncogene driver targeted agent. This potentiation effect of ATM inhibitor relies upon an enhanced activation of the apoptotic program as revealed by increased levels of BIM, cleaved-PARP, or cleaved-lamin A/C in response to combined treatment. Similar findings were obtained by silencing ATM using siRNA and treating NSCLC cells with oncogene driver inhibitors.

In previous studies, we showed that inhibition of oncogene drivers such as EGFR in NSCLC and BCR-ABL in CML caused a downregulation of glycolysis and an upregulation of OXPHOS [22, 23]. In the present article, when oncogene driver inhibitors were used in NSCLC cells silenced for ATM, we observed downregulation of OXPHOS indicating that ATM levels and its phosphorylation status are key factors for OXPHOS regulation. In other words, p-ATM integrates regulatory signals converging on mitochondria from different pathways determining the mitochondrial status and energy metabolism. In agreement with our observations, cells from subjects with ataxia-telangiectasia and knockout of ATM in mice and cells show mitochondrial dysfunction [27]. In particular, lack of ATM causes alterations in total mitochondrial DNA levels and mitochondrial mass and reduction of mitochondrial respiration rates [28–30]. Transcriptional activation of mitochondrial genes by nuclear respiratory factor 1 (NRF1) is reduced in ATM-deficient cells resulting in a decreased mitochondrial biogenesis [31]. An indirect regulation of mitochondrial homeostasis by ATM is reported to occur through histone H2AX, one of the primary targets of ATM following double-stranded DNA breaks. Loss of H2AX led to decrease of PGC-1α protein, a transcription coactivator that regulate the expression of OXPHOS, and consequently to reduced levels of subunits of the five OXPHOS complexes in both mouse embryonic fibroblasts and the brains of mutant mice [32]. Here, we showed that, independently from double-stranded DNA breaks, treatment with WZ4002 increases levels of p-ATM in H1975 cells and enhances the expression of PGC-1α, whereas the addition of even low doses of KU55933 prevents the upregulation of PGC-1α in response to TK inhibitors.

Furthermore, we showed that direct inhibition of ATM phosphorylation is able to reduce OXPHOS levels in oncogene-driven cancer cells and to enhance apoptosis in response to TK inhibitors, thus highlighting the possibility to use ATM inhibitors in combination therapy. However, in order to exploit this ability of ATM inhibitors in clinical studies, highly selective agents targeting only the noncanonical function of ATM are required to avoid the impairment of the canonical DNA repair function of ATM. Further studies are needed to identify targets downstream ATM that can be safely exploited for therapeutic purposes.

In conclusion, our study highlights the role of ATM in the maintenance of glycolytic phenotype despite functional mitochondria in oncogene-driven cancer cells since phosphorylated ATM is needed for the expression of glycolytic enzyme and OXPHOS. The lack of ATM or reduction of its phosphorylated form shift the glucose-dependent energy metabolism to other substrates and cause reduction of OXPHOS and mitochondrial dysfunction, thus rendering cancer cells more sensitive to oncogene driver inhibitors and apoptotic stimuli.

### Supplementary Information

**Additional file 1: Figure S1.** Expression levels of Glucose-6-phosphate dehydrogenase in H1993 and H1975 cells exposed to 100 nM KU55933 for 48 hours. Actin serves as equal loading control.

## Data Availability

All data generated and analyzed during this study are included in this published article and its supplementary information files.

## Abbreviations

AML: Acute myeloid leukemia
ATM: Ataxia-telangiectasia protein mutated
ATP: Adenosine triphosphate
AKT: Protein kinase B (PKB)
CML: Chronic myeloid leukemia
EGFR: Epidermal growth factor receptor
FLT3: FMS-like tyrosine kinase 3
GLUT: Glucose transporter type
G6PDH: Glucose-6-P dehydrogenase
HKII: Hexokinase
LDH: Lactate dehydrogenase
MET: Hepatocyte growth factor receptor
MRN: Mre11, Rad50, and Nbs1 complex
NSCLC: Non-small cell lung cancer
OD: Optical density
PDH: Pyruvate dehydrogenase complex
PK: Pyruvate kinase
PPP: Pentose phosphate pathway
siRNA: Small interfering RNA
TCA: Tricarboxylic acid
