# Supplementary figures and images for: ATM inhibition blocks glucose metabolism and amplifies the sensitivity of resistant lung cancer cell lines to oncogene driver inhibitors
Source: Cancer Metab. 2023 Nov 6;11:20. doi: 10.1186/s40170-023-00320-4 (PMC10629204; doi:10.1186/s40170-023-00320-4)

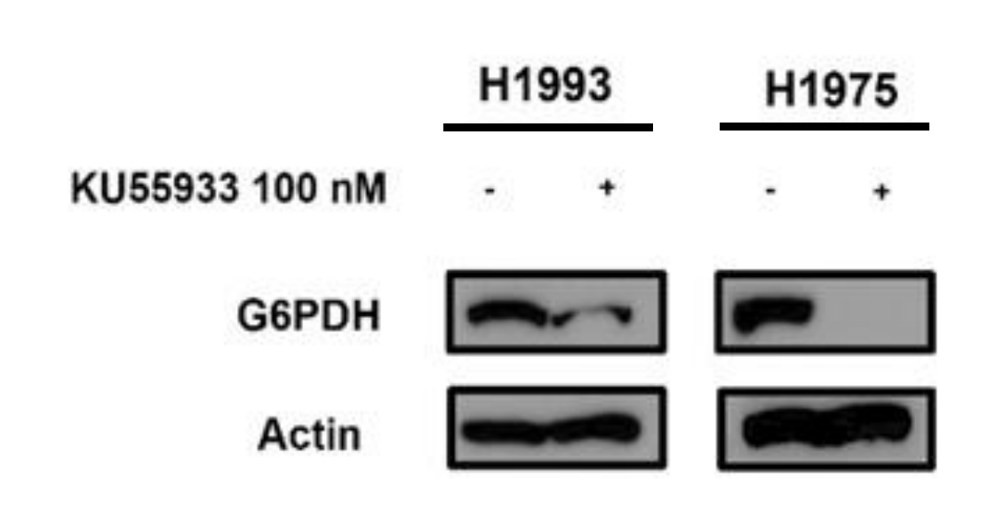

Supplement: Supplementary file 1 — Additional file 1: Figure S1. Expression levels of Glucose-6-phosphate dehydrogenase in H1993 and H1975 cells exposed to 100 nM KU55933 for 48 hours. Actin serves as equal loading control. [file 40170_2023_320_MOESM1_ESM.tif]
